# Molecular detection of virulence genes in *Salmonella* spp. isolated from chicken faeces in Mafikeng, South Africa

**DOI:** 10.4102/jsava.v91i0.1994

**Published:** 2020-07-21

**Authors:** Tsepo A. Ramatla, Nthabiseng Mphuthi, Taole Ramaili, Moeti O. Taioe, Oriel M.M. Thekisoe, Michelo Syakalima

**Affiliations:** 1 Department of Animal Health, Faculty of Natural and Agricultural Sciences, North-West University, Mafikeng, South Africa; 2 National Zoological Garden, South African National Biodiversity Institute, Pretoria, South Africa; 3 Unit for Environmental Sciences and Management, Faculty of Natural and Agricultural Sciences, North-West University, Potchefstroom, South Africa

**Keywords:** *Salmonella* spp., virulence genes, chicken, South Africa, microbiology

## Abstract

Chickens have been implicated in most *Salmonella* disease outbreaks because they act as carriers of the pathogen in their gut. There are over 2500 serotypes of *Salmonella* that have been reported worldwide and 2000 of these serovars can be found in chickens. The main objective of this study was to determine the *Salmonella* serotypes found in poultry farms around Mafikeng district, South Africa. *Salmonella* was identified according to the guidelines of the International Organization for Standardization (ISO) (ISO 6579:2002) standard techniques. Faecal samples were collected and analysed for *Salmonella* using conventional cultural methods and polymerase chain reaction targeting the *16S Ribosomal Deoxyribonucleic acid (rDNA)* gene for *Salmonella* identification. Out of 130 presumptive *Salmonella* isolates determined by urease and triple sugar iron tests, only 46 isolates were identified as *Salmonella* serotypes of which *S*. Typhimurium was the most frequent with 18 (39.1%), followed by *S*. Heidelberg with 9 (19.6%), *S. bongori* with 7 (15.2%), *S*. Enteritidis with 6 (13.0%) and both *S.* Paratyphi B and *S*. Newport with 3 (6.5%) each. Seven virulence genes including *inv*A 100%, *spy* 39%, *hil*A 9%, *mis*L 30%, *sdf*I 13%, *orf*L 11% and *spi*C 9% were detected from these *Salmonella* isolates in this study. The presence of these virulence genes indicates high pathogenicity potential of these isolates which is a serious public health concern because of zoonotic potential of *Salmonella*.

## Introduction

Commercial poultry production is rapidly growing worldwide to meet the needs of the increasing human population (Olobatoke & Mulugeta 2015). Many poor and middle-class farmers in developing countries are taking up poultry to supplement their income, and this industry has become a major income source to them. Therefore, the presence of pathogenic organisms such as *Salmonella* in chickens, as a major food-borne infection in humans, can have an adverse impact on the production and marketing of poultry (Imanishi et al. 2015). Chickens have been implicated in most of *Salmonella* outbreaks because they act as carriers of this pathogen in their guts (Black 2008).

*Salmonella* that can be traced through chickens can be classified into three groups (Hafez 2013). The first group includes highly host adapted and invasive serotypes such as *S. typhi* in humans and *S. gallinarum* and *S. pullorum* in poultry. The second group is non-host-adapted and invasive serotypes such as *S.* Typhimurium, *S.* Arizonae and *S.* Enteritidis. The third group contains non-host-adapted and non-invasive serotypes, and most of these serotypes are harmless to animals and humans (Andino & Hanning 2015; Umali et al. 2012). Understanding the mechanisms of *Salmonella* infection, intestinal colonisation, persistence and excretion in poultry are essential to discover appropriate measures to decrease both contamination of flocks and public health risk (Andino & Hanning 2015).

Some of these *Salmonella* spp. can carry or harbour virulence genes (Peixoto et al. 2017) responsible for important characteristics such as intracellular survival (Pathmanathan et al. 2003), as well as the production of Vi capsular antigens and cell invasion (Peixoto et al. 2017). Several virulence genes in *Salmonella* are known, and most are situated in *Salmonella* pathogenicity islands (SPIs), prophages, plasmids and fimbrial clusters (Prasanna Kumar 2016). However, the majority of the virulence genes are gathered within SPI-1 and SPI-2 (Marcus et al. 2000). The SPI-1 encodes factors vital for cell adhesion, while SPI-2 encodes factors essential for replication and intracellular survival (Majowicz et al. 2010). The SPIs play important roles in invasion, antibiotic resistance, adhesion, systemic infection, fimbrial expression, toxin production, intracellular survival, and Mg^2+^ and iron uptake (Kim & Ju Lee 2017; Majowicz et al. 2010).

The role played by the virulence genes of *Salmonella* species, are known based on observations made on epithelial cells. The *inv*A gene for instance affects the host cell by delivery of type III secreted effectors, for mutant phenotype, and is also essential for invasion of epithelial cells (El-Sharkawy et al. 2017; Marcus et al. 2000). The *inv*A gene has been confirmed to be present in *Salmonella* species only and hence is used in the genetic diagnosis of *Salmonella* species (Fekry, Ammar & Hussien 2018).

The operon *spv* (*Salmonella* plasmid virulence) is considered as one of the virulence plasmids of numerous *Salmonella* serotypes that generate systemic disease (Castilla et al. 2006). It harbours five genes *spv*RABCD (Rotger & Casadesús 1999) that have been identified to contribute to its pathogenicity (Card et al. 2016; Haneda et al. 2012). The presence of *Hil*A gene in *Salmonella* is essential for the expression of the type III secretion system (TTSS) components, and it encodes the central regulator *Hil*A (Borges et al. 2013). This gene (*Hil*A) is required to induce apoptosis of macrophages and invade epithelial cells (Borges et al. 2013). The *sip*C gene acts as a translocase, mediating bacterial entry into epithelial (Prasad 2012). On the other hand, *spi*C acts to modulate invasion gene expression (Hayward & Koronakis 1999).

The main objective of this study was to detect prevalent *Salmonella* serotypes in chicken faeces from poultry farms around Mafikeng in South Africa and assess the presence of virulence genes using conventional polymerase chain reaction (PCR) assays.

## Materials and methods

### Sampling site

**FIGURE 1:**
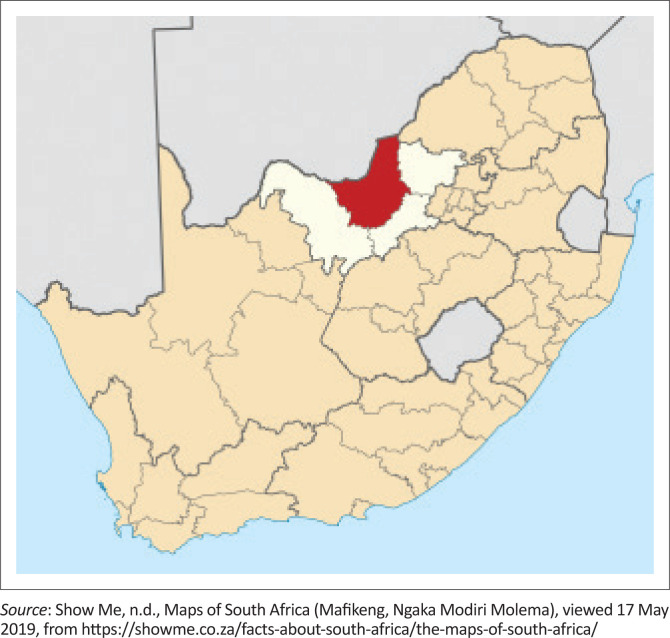
Map of South Africa, the red part, shows Mafikeng within the North West province.

The study area covered Mafikeng in North West Province, South Africa, as shown in Figure 1. The province is the second largest chicken producer in South Africa at 21.3% after the Western Cape with 21.9%. The longitude and latitude of the district are 25°50′E and 25°55′N, respectively. Temperatures range from 3 °C to 21 °C in the winter and from 17 °C to 31 °C in the summer. The average rainfall is 360 mm.

### Sample collection

A list of poultry farms in the Mafikeng area was compiled using the records of the provincial Department of Agriculture of North West provincial government. A few farms in the south, east, north and west were randomly selected, the farmers were approached and those who agreed were included in the study. Sampling sites were therefore conveniently selected. Sterile spatulas were used to collect samples of freshly passed poultry droppings in sterile universal sampling bottles.

Samples were collected at three different points in each poultry farm once a week. This was performed to have a good representation and distribution of the organisms. After collection, samples were packed in properly labelled sterile polyethylene bags and transported in a sterilised icebox and processed immediately upon arrival to the laboratory.

### Isolation and identification of *Salmonella*

Each faecal sample (1 g) was weighed and transferred into a sterile container. A volume of 10 mL of buffered peptone water (BPW Oxoid, Biolab, South Africa) was added into each sample and then homogenised by vortexing for about 2 minutes followed by incubation at 37 °C ± 1 °C for 18 hours - 24 hours Thereafter, 1 mL of the sample was transferred to 10 mL of Mueller–Kauffmann Tetrathionate Novobiocin (MKTTn) broth (Sigma-Aldrich, S.A. Barcelona, Spain) which was incubated at 4.5 °C for 6 h. Then, 1 mL was transferred from MKTTnB to 10 mL of Rappaport–Vassiliadis medium with soya (RVS) broth (Sigma-Aldrich, S.A. India) and incubated at 36 °C for 24 h. A 1 mL aliquot from the RVS broth was then transferred to a 30% glycerol solution (EMD chemicals, United States [US]) and stored at −20 °C for later use.

### Culturing of *Salmonella* on selective agar plates

After incubation, a loopful of the enriched cultures of RVS broth was streaked separately onto two selective agar plates: Xylose Lysine Deoxycholate agar (XLD) (Merck, Wadeville, South Africa) and Brilliant Green Agar (BGA) (Scharlau Chemie S.A. Barcelona). These plates were incubated in an overturned position at 37 °C ± 1 °C for 18 h – 24 h. Following incubation, the black and pink colonies with or without black centre on XLD agar, the colourless or opaque white colonies surrounded by pink or red zone and the red colonies on BGA were identified as suspected *Salmonella.* Suspected colonies of *Salmonella* spp. were then confirmed according to the guidelines of ISO 6579: 2002. Such colonies were picked out and streaked on Nutrient agar (NA) (Merck, Wadeville, South Africa) and incubated at 37 °C ± 1 °C for 18 h - 24 h.

### Biochemical identification

All gram-negative, rod-shaped bacteria were subjected to preliminary biochemical tests. The presumptive identification of *Salmonella* colonies was carried out using urease and triple sugar iron (TSI) tests. Two to three purified colonies of the presumptive *Salmonella* isolates from the 24-h NA plates were subjected to the urea broth and incubated at 37 °C for 18 h – 24 h. Presumptive *Salmonella* colonies were also carried out by stabbing the butt of TSI slants, and the slants were incubated at 37 °C and examined after 18 h – 24 h for gas production, hydrogen sulphide production and carbohydrate fermentation. The analytical profile index (API) 20E (BioMerieux, Marcy l’Etoile, France) was used for identification, and indices were generated for the diverse isolates and used to verify their identities using the API web^TM^ identification software. Biochemical identification was performed in order to get presumptive positives and as part of a large set of other experiments that were being conducted on the samples.

### Genomic deoxyribonucleic acid extraction

Genomic deoxyribonucleic acid (DNA) was extracted using Fungal/Bacterial Soil Microbe DNA Mini Prep kit according to the manufacturer’s instructions (Zymo-Research, US). Extracted DNA was eluted with 100 *µ*L of DNA elution buffer into clean 1.5 mL micro-centrifuge tube and stored at −20 °C for molecular confirmation of the *Salmonella* species and detection of virulence genes.

### Molecular identification of *Salmonella* serovars using *16S rDNA* gene

Polymerase chain reactions were conducted using the Universal primers: forward (5’-AGA GTT TGA TCC TGG CTC AG-3’) and the Reverse (5’-ACG GCT ACC TTG TTA CGA CTT-3’), with the reaction volume of 25 *µ*L containing 12.5 *µ*L PCR Master Mix, 2 *µ*L template DNA, 8.5 *µ*L nuclease free water and 1 *µ*L of each oligonucleotide primer using an Engine T100 Thermal^TM^ cycler (Bio-Rad, Singapore). The thermo cycling conditions consisted of an initial denaturation step at 95 °C for 5 min followed by 35 cycles of denaturation at 94 °C for 30 s, annealing at 61 °C for 30 s and extension at 72 °C for 5 min, and finally a single and final extension step at 72 °C for 7 min. Polymerase chain reaction products were identified by electrophoresis on 2% (weight per volumne [w/v]) agarose gel stained with ethidium and visualised under ultraviolet light on a ChemiDoc Imaging System (Bio-Rad ChemiDocTM MP Imaging System, Hertfordshire, United Kingdom).

### Deoxyribonucleic acid sequencing

Amplified PCR products were sequenced using an ABI PRISM® 3500XL DNA Sequencer (Applied Biosystems) at Inqaba Biotechnical Industrial (Pty) Ltd. (Pretoria, South Africa). The acquired sequences were aligned on GenBank database using Basic Local Alignment Search Tool (BLAST) (www.ncbi.nlm.nih.gov/BLAST) from the National Center for Biotechnology Information (NCBI) to identify sequences with high similarity.

### Detection of virulence genes using polymerase chain reaction

Seven pairs of published oligonucleotide primers were used to detect the virulence genes using PCR. The individual PCRs, for each virulent gene, were set up in a 25 *µ*L, which consisted of 12.5 *µ*L AmpliTaq Gold® 360 PCR Master Mix (AmpliTaq Gold® DNA Polymerase 0.05 units/*µ*L, Gold buffer 930 mM Tris/HCl pH 8.05, 100 mM KClO, 400 mM of each dNTP and 5 mM MgCl_2_) (Applied Biosystems, California, US). Then, 2.5 mM of each primer, 2 *µ*L of template DNA and ddH_2_O were added to make the final volume. Test DNA was replaced with 5 *µ*L of sterile nuclease-free water as negative control. Cycling conditions for PCR as well as the information of the published primers used are detailed in Table 1.

**TABLE 1:** Primers sequences, source of primers for amplification and polymerase chain reaction conditions used for in this study.

Target gene	Sequence (5’–3’)	Amplicon size (bp)	Conditions	References
*inv*A	GTGAAATTATCGCCACGTTCGGGCAA TCATCGCACCGTCAAAGGAACC	280	94 °C for 5 min, 94 °C for 45 s, 58 °C for 45 s, 72 °C for 70 min, 72 °C for 7 min, 30 cycles	Ekwanzala et al. 2017
*SdfI*	TGTGTTTTATCTGATGCAAGAGG TGAACTACGTTCGTTCTTCTGG	303	95 °C for 2 min, 95 °C for 1 min, 57 °C for 1 min, 72 °C for 2 min; 72 °C for 5 min, 30 cycles	Mohd Afendy & Son 2015
*Spy*	TTGTTCACTTTTTACCCCTGAA CCCTGACAGCCGTTAGATATT	401	95 °C for 2 min, 95 °C for 30 s, 57 °C for 30 s, 72 °C for 30 s, 72 °C for 4 min, 30 cycles	Alvarez et al. 2004
*hil*A	CGGAACGTTATTTGCGCCATGCTGAGGTAG GCATGGATCCCCGCCGGCGAGATTGTG	784	94 °C, 5 min, 94 °C for 1 min, at 65 °C for 1 min, 72 °C for 1 min, 72 °C for 10 min, 30 cycles	Modarressi & Thong 2010
misL	GTCGGCGAATGCCGCGAATA GCGCTGTTAACGCTAATAGT	400	94 °C for 3 min, 94 °C for 1 min, 58 °C for 1 min, 72 °C for 1 min, 72 °C for 5 min, 35 cycles	Zishiri, Mkhize & Mukaratirwa 2016
orfL	GGAGTATCGATAAAGATGTT GCGCGTAACGTCAGAATCAA	550	94 °C for 3 min, 94 °C for 1 min, 58 °C for 1 min, 72 °C for 1 min, 72 °C for 5 min, 35 cycles	Zishiri et al. 2016
spiC	CCTGGATAATGACTATTGAT AGTTTATGGTGATTGCGTAT	309	94 °C for 3 min, 94 °C for 1 min, 55 °C for 60 min, 72 °C for 60 min, 72 °C for 5 min, 30 cycles	Zishiri et al. 2016

bp, base pairs; min, minute; s, second; h, hour.

### Phylogenetic analysis

All confirmed sequencing results were edited by using Bio-Edit software (Hall 1999) and saved as FASTA format. The sequences were used to search the GenBank database with the BLASTn algorithm to find out the relative Phylogenetic positions. The sequences were aligned by using the multiple alignment fast Fourier transform (MAFFT) programme 6.8464 to conduct multiple and pairwise sequence alignments against corresponding nucleotide sequences retrieved from GenBank. Evolutionary distance matrices were generated as described previously by Jukes and Cantor (1969). Phylogenetic analysis was performed using MEGA version 7 (Kumar, Stecher & Tamura 2016), and neighbour-joining (NJ), maximum-parsimony, and maximum likelihood methods were used for the construction of the trees. Bootstrap analyses were performed using 1000 replications for NJ, maximum-parsimony, maximum likelihood. Recognised chimeric sequences were identified using the Chimera Buster 1.0 software. Manipulation and tree editing were carried out using Tree View (Timme et al. 2013).

### Statistical analysis

Descriptive statistics, particularly proportions, percentages, means and standard deviations, were determined using Excel software (2013).

### Ethical considerations

Prior to the commencement of the study, the research proposal was approved based on Animal Research Ethics Committee (NWU-00274-18-A5) guidelines by North-West University Research Ethics Regulatory Committee (NWU-RERC).

## Results

### Prevalence of *Salmonella* infection in chicken’s droppings

Out of 130 presumptive *Salmonella* isolates, only 46 (35.4%) isolates were identified as *Salmonella* species after blasting to identify sequences with high similarity. The predominant *Salmonella* serovars isolated were *S.* Typhimurium 18 (39.1%), *S.* Heidelberg 9 (19.6%), *S. bongori* 7 (15.2%), *S.* Enteritidis 6 (13.0%), *S. enterica* Paratyphi B 3 (6.5%) and *S.* Newport 3 (6.5%). The sequences obtained from this study are accessible from GenBank database and were given accession numbers, as shown in Table 2.

**TABLE 2:** Results of 46 isolates for analytical profile index, *16S Ribosomal ribonucleic acid* sequencing (polymerase chain reaction) and accession number.

Isolates ID	Sequence length (bp)	*16S rRNA* results	API identification	Accession number
C1	971	*Salmonella* Enteritidis	*Salmonella* spp.	MH356670.1
C2	1057	*Salmonella* Heidelberg	+	MH356671.1
C3	1059	*Salmonella* Paratyphi B	+	MH356672.1
C4	979	Salmonella bongori	+	MH356673.1
C5	1009	*Salmonella* Newport	+	MH356674.1
C6	1029	*Salmonella* Typhimurium	+	MH356675.1
C7	979	*Salmonella* Typhimurium	+	MH356676.1
C8	1034	*Salmonella* Enteritidis	+	MH356677.1
C9	1060	*Salmonella* Newport	+	MH356678.1
C10	987	*Salmonella* Paratyphi B	+	MH356679.1
C11	994	*Salmonella* Typhimurium	+	MH356680.1
C12	1080	*Salmonella* Typhimurium	+	MH356681.1
C13	1049	Salmonella bongori	+	MH356682.1
C14	1051	*Salmonella* Heidelberg	*Salmonella* spp.	MH356683.1
C15	1074	*Salmonella* Heidelberg	+	MH356684.1
C16	1058	*Salmonella* Paratyphi B	+	MH356685.1
C17	1019	Salmonella bongori	+	MH356686.1
C18	1069	*Salmonella* Newport	+	MH356687.1
C19	1019	*Salmonella* Heidelberg	+	MH356688.1
C20	979	*Salmonella* Enteritidis	+	MH356689.1
C21	971	Salmonella bongori	+	MH356690.1
C22	1025	*Salmonella* Enteritidis	*Salmonella* spp.	MH356691.1
C23	1050	*Salmonella* Typhimurium	+	MH356692.1
C24	1049	Salmonella bongori	+	MH356693.1
C25	1012	*Salmonella* Heidelberg	+	MH356694.1
C26	989	*Salmonella* Typhimurium	+	MH356695.1
C27	1062	*Salmonella* Heidelberg	+	MH356696.1
C28	979	*Salmonella* Typhimurium	+	MH356697.1
C29	709	*Salmonella* Enteritidis	+	MH356698.1
C30	1056	*Salmonella* Typhimurium	*Salmonella* spp.	MH356699.1
C31	895	Salmonella bongori	+	MH356700.1
C32	1077	*Salmonella* Typhimurium	+	MH356701.1
C33	1087	Salmonella bongori	+	MH356702.1
C34	1007	*Salmonella* Typhimurium	+	MH356703.1
C35	1034	*Salmonella* Typhimurium	+	MH356704.1
C36	868	*Salmonella* Typhimurium	+	MH356705.1
C37	1069	*Salmonella* Typhimurium	+	MH356706.1
C38	999	*Salmonella* Heidelberg	*Salmonella* spp.	MH356707.1
C39	1057	*Salmonella* Heidelberg	+	MH356708.1
C40	938	*Salmonella* Enteritidis	+	MH356709.1
C41	848	*Salmonella* Typhimurium	+	MH356710.1
C42	925	*Salmonella* Typhimurium	*Salmonella* spp.	MH356711.1
C43	721	*Salmonella* Typhimurium	+	MH356712.1
C44	986	*Salmonella* Typhimurium	+	MH356713.1
C45	933	*Salmonella* Typhimurium	+	MH356714.1
C46	1061	*Salmonella* Typhimurium	+	MH356715.1

rRNA, *Ribosomal ribonucleic acid;* API, analytical profile index; bp, base pairs.

### Phylogenetic analysis of the *Salmonella* isolates from chickens

**FIGURE 2:**
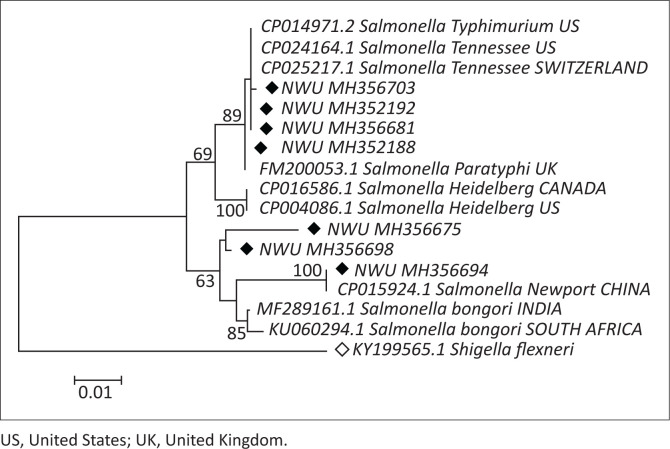
The phylogenetic relationship of *Salmonella* detected in the faeces from chickens. The tree was clustered with the neighbour-joining method using MEGA 7 package. Bootstrap values based on 1000 replications were listed as percentages at the nodes. The GenBank accession numbers are indicated at the branches of the tree. The scale bar indicates 0.01 substitutions per nucleotide position. Bootstrap values > 60% are shown.

A phylogenetic tree for *Salmonella* isolates from chickens was constructed to understand the genetic closeness of *Salmonella* strains with other related strains from different countries inside and outside the African continent. *Shigella fleneri* (KY199565) from the family Enterobacteriaceae was used as an out-group for the *16S-Ribosomal Deoxyribonucleic acid* gene. The resulting NJ revealed that strain *S.* Heidelberg clustered closely with sequences of the following *S.* Heidelberg: CP004086.1, originating from retail meats, humans and animals; CP016586.1, originating from food sources, human and animal; *S.* Tennessee: CP024164, originating from laboratory control strains and CP025217.1, originating from the food industry; *S. bongori*: MF289161.1, originating from the crops, and MG663480.1, originating from chicken faeces; *S. enterica* Paratyphi; CP006575.1, originating from bacteria strain obtained from source laboratory of Zoonosis; *S.* Typhimurium: CP014971.2, originating from humans and cattle; *S.* Enteritidis: CP018651.1, originating from the historical *S.* Enteritidis isolated between the 1940s and 1990s. The phylogenetic tree is shown in Figure 2.

### Distribution of virulence genes among *Salmonella* isolates

A representative detection of *Salmonella* virulence genes is shown in Table 3. The results revealed that 100% isolates were harbouring *inv*A gene, 39% harboured *spy* gene, 9% harboured *hil*A gene, 30.1% harboured *mis*L gene and 13% harboured *sdf*I gene, while 11% harboured *orf*L gene and only 9% harboured *spi*C gene.

**TABLE 3:** Prevalence of detected virulence genes from *Salmonella* isolates.

Isolates	*invA*	*Spy*	*hilA*	*misL*	*Sdf I*	*orfL*	*spiC*
*Salmonella* Typhimurium	18	18	-	7	-	2	1
*Salmonella* Enteritidis	6	-	1	2	6	1	1
*Salmonella* Newport	3	-	-	-	-	-	-
*Salmonella* Heidelberg	9	-	1	2	-	-	-
Salmonella Bongori	7	-	1	2	-	2	1
*Salmonella enterica* serovar Paratyphi B	3	-	1	1	-	-	1

**Total**	**46**	**18(39%)**	**4(9%)**	**14(30%)**	**6(13%)**	**5(11%)**	**4(9%)**

## Discussion

The primary objective of this study was to isolate and detect the types of *Salmonella* spp. from chicken droppings around Mafikeng, South Africa. Six *Salmonella* serovars were isolated, namely *S.* Typhimurium, *S.* Heidelberg, *S. bongori, S.* Enteritidis, *S.* Paratyphi B and *S.* Newport. All these serovars are known to be pathogenic in humans and cause salmonellosis (Fagbamila et al. 2017; Orji, Onuigbo & Mbata 2005; Tejada et al. 2016). Among the *Salmonella* isolates detected from this study, *S.* Typhimurium, *S.* Enteritidis and *S.* Newport have previously been reported in raw broiler samples by another study here in Mafikeng, South Africa (Olobatoke & Mulugeta 2015). The presence of these isolates, especially *S.* Typhimurium and *S.* Enteritidis as determined by this study, is a concern because they are known to cause serious human illness (Alvarez et al. 2004; Lapuz et al. 2008). A previous study of *Salmonella* isolates in South Africa undertaken by Kidanemariam, Engelbrecht and Picard (2010) between 1999 and 2006 revealed the 10 common *Salmonella* serotypes isolated: *S.* Newport, *S.* Typhimurium, *S.* Dublin, *S*. Enteritidis, *S.* Muenchen, *S.* Chester, *S*. Heidelberg, *S.* Hadar, *S.* Schwarzengrund and *S*. Mbandaka. Our study did not isolate all these *Salmonella* species in the Mafikeng chicken samples but agrees with that study which identifies *S.* Typhimurium as the most prevalent isolate in poultry (Kidanemariam et al. 2010).

Many poor and middle-class farmers in developing countries are taking up poultry to supplement their income, and this industry has become a major income source to them. Unfortunately, there has also been an increase in human salmonellosis cases, and these have been linked with consumption of contaminated chicken products (Imanishi et al. 2015; Lebert et al. 2018; Olobatoke & Mulugeta 2015). Four of the serotypes detected in this study, namely *S.* Typhimurium, *S.* Enteritidis, *S.* Newport and *S.* Heidelberg, are in the top five most frequently isolated serotypes in poultry meat and in human disease in South Africa and elsewhere as indicated by the phylogenetic analysis. For example, *S.* Heidelberg was associated with the 2011 outbreak, *Salmonella* outbreak, in South Africa (Kidanemariam et al. 2010); *S.* Typhimurium was responsible for salmonellosis outbreak in Australia and in Germany from 2001 to 2005 (De Freitas Neto et al. 2010) and 63 *Salmonella* outbreaks in Italy (De Freitas Neto et al. 2010). Additionally, the majority of *Salmonella* cases worldwide are caused by *S.* serovar Enteritidis, from eggs and poultry meat (Backhans & Fellström 2012). *Salmonella enterica* serovar Paratyphi B has been linked with human outbreaks in the different countries like US (Harris et al. 2009), Australia (Levings et al. 2006), Canada (Stratton et al. 2001) and European countries (Miko et al. 2002). This is noteworthy as it highlights the possibility that the detected strains from this study may also play a significant role in human disease here in the province if proper sanitary measures are not applied. The phylogenetic analysis also revealed that the *Salmonella* isolates found in this study are very similar to other genotypes previously identified in several other sources, including humans, milk, chickens and other animals. This therefore indicates that the strains that were found in chickens could easily be circulating in other poultry, livestock, animal products and/or humans.

The disease causing potential of *Salmonella* isolates is derived from the virulent genes that they may carry. So, this study undertook to determine the presence of these virulent genes to understand how potentially pathogenic the strains were (Ekwanzala et al. 2017; Sunar et al. 2014). Forty-six (*n* = 46) *Salmonella* isolates were analysed for the presence of seven known virulence genes, namely *inv*A, *Sdf*I, *hil*A, *misL, Spy, orfL* and *spiC*. It was interesting to note that all the tested virulence genes were detected by this study in differing proportions. All 46 (*n* = 46) isolates were positive for *inv*A, indicating that all of them have the ability to invade and to cause gastroenteritis (Ekwanzala et al. 2017; Hu et al. 2008; Lan et al. 2018; Odjadjare & Olaniran 2015; Sunar et al. 2014) and can survive in macrophages (Goodman et al. 2017). The inner membrane of *Salmonella* contains protein coded for by invA, which is vital for invasion into epithelial cells (Salehi, Mahzounieh & Saeedzadeh 2005). This gene (*inv*A) is found in Pathogenicity island-1 and it is a TTSS apparatus, which secretes invasion effectors like invasion factor A (Sabbagh et al. 2010). Eighteen isolates harboured *spy*, and these isolates were identified as those of *S.* Typhimurium. This gene has been used to identify and confirm *S.* Typhimurium (Can et al. 2014). The *spy* gene appears to exclusively act as a molecular chaperone (Wells 2015) and thus confers pathogenicity to this strain. The *Sdf I* gene has been used to identify *S.* Enteritidis (Mohd Afendy & Son 2015). *Salmonella* serovars encoding *spy* and *Sdf I* (*S.* Typhimurium and *S.* Enteritidis) are known to be associated with human illness (Odjadjare & Olaniran 2015).

Out of 46 *Salmonella* isolates, 14 were identified as harbouring the *misL* gene. This gene aids virulence by being involved in the intra-macrophage survival of the pathogen (Hughes et al. 2008). The *misL* gene is located in the SPI-3 of *Salmonella* (Zishiri et al. 2016). The *orfL* and *spiC* genes were positive from five and four isolates, respectively. The *spi*C gene is one of the virulence factors of *Salmonella* which is found in island 2 and associated with type 3 effector protein. It normally disrupts the vesicular transport of the host cell (Kaur & Jain 2012). The *orfL* gene, on the other hand, has a secretion system that mediates the secretion of toxins and is necessary for macrophages survival (Odjadjare & Olaniran 2015).

The presence of these genes in the isolates from Mafikeng therefore shows that these strains are of public health importance and can cause disease when and if contamination is not properly managed.

## Conclusion

This study has identified *Salmonella* isolates that have traditionally been associated with disease not only in South Africa but elsewhere in the world. These isolates have the virulent genes that are important for pathogenesis and therefore are of serious public health concern and measures should be put to control disease outbreaks in Mafikeng specifically and South Africa in general.
